# Eos Negatively Regulates Human γ-globin Gene Transcription during Erythroid Differentiation

**DOI:** 10.1371/journal.pone.0022907

**Published:** 2011-07-28

**Authors:** Hai-Chuan Yu, Hua-Lu Zhao, Zhi-Kui Wu, Jun-Wu Zhang

**Affiliations:** 1 National Laboratory of Medical Molecular Biology, Institute of Basic Medical Sciences, Chinese Academy of Medical Sciences and Peking Union Medical College, Beijing, China; 2 Molecular Biology Laboratory of Guang'anmen Hospital, China Academy of Chinese Medical Sciences, Beijing, China; Southern Illinois University, United States of America

## Abstract

**Background:**

Human globin gene expression is precisely regulated by a complicated network of transcription factors and chromatin modifying activities during development and erythropoiesis. Eos (Ikaros family zinc finger 4, IKZF4), a member of the zinc finger transcription factor Ikaros family, plays a pivotal role as a repressor of gene expression. The aim of this study was to examine the role of Eos in globin gene regulation.

**Methodology/Principal Findings:**

Western blot and quantitative real-time PCR detected a gradual decrease in Eos expression during erythroid differentiation of hemin-induced K562 cells and Epo-induced CD34+ hematopoietic stem/progenitor cells (HPCs). DNA transfection and lentivirus-mediated gene transfer demonstrated that the enforced expression of Eos significantly represses the expression of γ-globin, but not other globin genes, in K562 cells and CD34+ HPCs. Consistent with a direct role of Eos in globin gene regulation, chromatin immunoprecipitaion and dual-luciferase reporter assays identified three discrete sites located in the DNase I hypersensitivity site 3 (HS3) of the β-globin locus control region (LCR), the promoter regions of the Gγ- and Aγ- globin genes, as functional binding sites of Eos protein. A chromosome conformation capture (3C) assay indicated that Eos may repress the interaction between the LCR and the γ-globin gene promoter. In addition, erythroid differentiation was inhibited by enforced expression of Eos in K562 cells and CD34+ HPCs.

**Conclusions/Significance:**

Our results demonstrate that Eos plays an important role in the transcriptional regulation of the γ-globin gene during erythroid differentiation.

## Introduction

The human β-globin locus consists of five functional globin genes (ε, Gγ, Aγ, δ, and β) within a 70 kb domain. During development expression of these genes displays two switches, the embryonic (ε-) to fetal (Gγ- and Aγ-) globin switching, coinciding with the transition from yolk sac to fetal liver, and the fetal to adult (β-) globin switching, occurring near the parturient period with the establishment of bone marrow as the main site of hematopoiesis [1], [2]. During erythroid differentiation the γ- to β-gloin gene switching is also displayed and it is called “compressed switching” [3]. The precise developmental program of human β-like globin gene expression is governed by a diverse array of regulatory mechanisms. Sequences within or immediately flanking globin genes control expression in tissue-specific or temporal patterns. High-level globin expression is directed by the locus control region (LCR), a set of key regulatory sequences 6–20 kb upstream of the ε-globin gene, that are characterized by the presence of five 5′ DNase I hypersensitivity sites (HSs) [4]. Preferential interactions between the LCR and individual globin promoters during distinct developmental stages are pivotal to the strict regulation of globin gene expression. These interactions are mediated by erythroid tissue-restricted and ubiquitous transcription factors.

Because fetal γ-globin gene reactivation in adults has potential as an effective therapy for sickle cell anemia and β-thalassemia [5], the detailed characterization of γ-globin gene regulation mechanisms is particularly significant. Several studies have reported transcriptional activation of the γ-globin gene by FKLF [6], FKLF2 [7], NF-E4 [8] and NF-Y [9]. However, repressors also play a critical role during γ- to β-globin switching. The repressors BCL11A [10], Ikaros [11], GATA-1 [12], the orphan nuclear receptors TR2 and TR4 [13], and NF-E3/COUP-TFII [14] have been associated with human γ-globin gene silencing. Despite avid research regarding γ-globin gene regulation, the mechanisms responsible for γ-globin gene silencing are not fully understood.

Eos, also known as IKZF4, is a member of the zinc finger transcription factor Ikaros family characterized by the presence of four DNA-binding N-terminal zinc fingers and two C-terminal zinc fingers required for homo- and heterodimerization with other Ikaros family members [15]. Ikaros family of genes consists of several members: Ikaros (IKZF1), Aiolos (IKZF3), Helios (IKZF2), Eos (IKZF4) and Pegasus (IKZF5). The Ikaros family of transcription factors acts as key repressors of transcription during the development and function of lymphocytes [16]–[19]. Ikaros is involved in regulation of human β-like globin gene switching by binding to critical cis elements implicated in the gene switching and facilitating long-distance DNA looping between the LCR and a region upstream of δ-globin. When the DNA-binding region of Ikaros is disrupted by a point mutation in plastic mice, concomitant marked downregulation of β-globin expression and upregulation of γ-globin expression are observed [20]. Eos is a 585 amino acid highly conserved zinc finger transcription factor that binds typical WGGGAAT Ikaros recognition sites in DNA and functions as a transcriptional repressor (Figure S1) [21]. Eos may also play an important role in the development of the central and peripheral nervous systems [22], [23]. Eos can self-associate, form heterodimers with other Ikaros family members, or interact with C-terminal binding protein (CtBP2), PU.1, or microphthalmia-associated transcription factor (MITF) to repress transcription of cathepsin K and tartrate-resistant acid phosphatase (TRAP) promoters [21], [24]. Eos is expressed at low levels in kidney, thymus, liver and heart and at high levels in skeletal muscle [23]. Eos mediates Foxp3-dependent gene silencing in CD4+ regulatory T cells by interacting directly with Foxp3 and inducing chromatin modifications that result in gene silencing in CD4+ regulatory T cells [19]. Although it has known that Eos protein is expressed in lymphocytes and is implicated in the control of lymphoid cell development, the function of Eos in the regulation of other haemopoietic lineage has not been addressed.

In this study, we examined the effects of Eos on human globin gene regulation and demonstrated its important role in γ-globin gene regulation during erythroid differentiation.

## Results

### Eos represses γ-globin gene expression in K562 cells during erythroid differentiation

**Figure 1:**
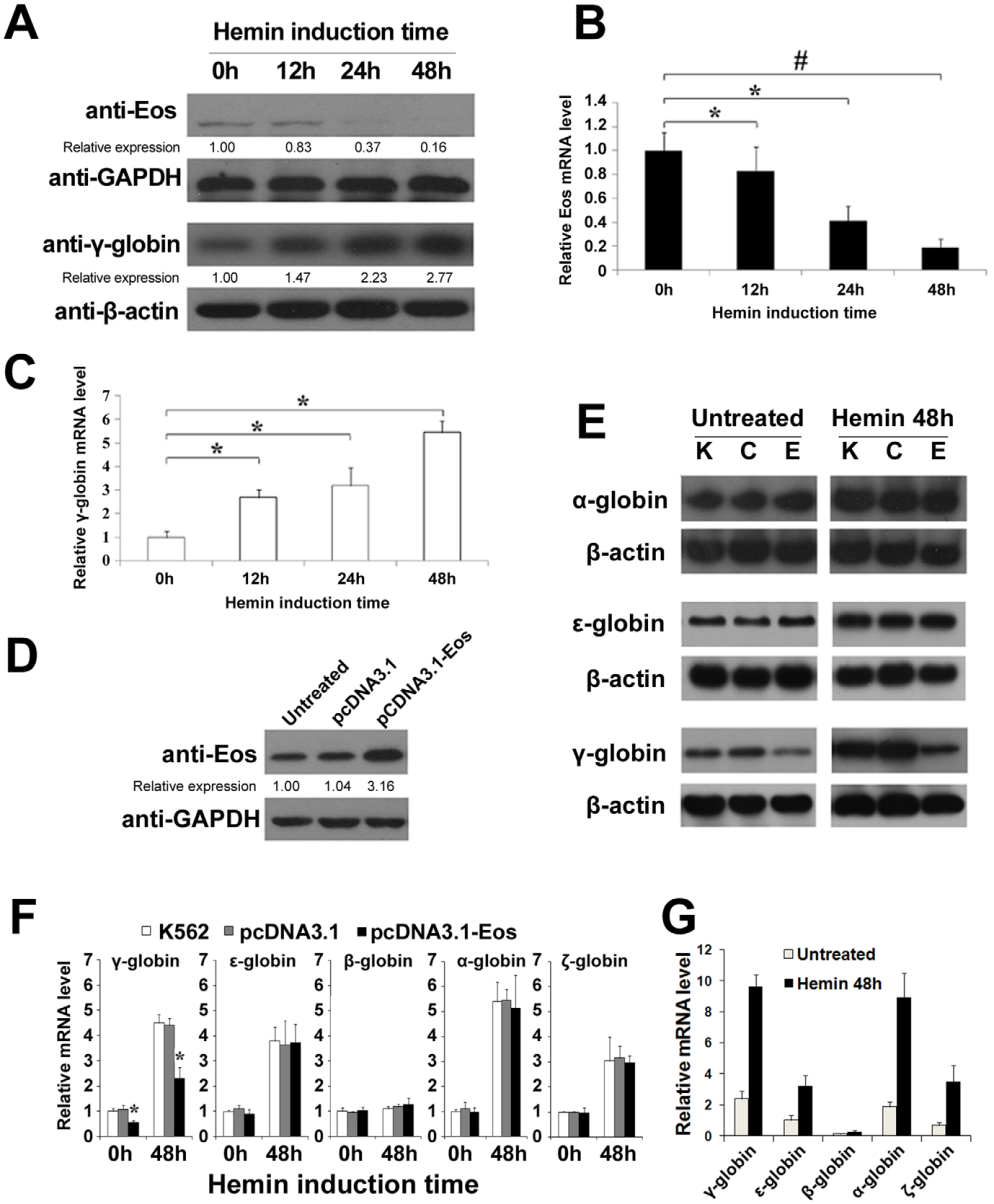
Eos represses γ-globin gene expression specifically in K562 cells. (**A**) Western blot analysis indicated a decrease in Eos protein levels and a marked increase in γ-globin levels during erythroid differentiation of hemin-induced K562 cells. (**B–C**) Quantitative real-time PCR analysis of Eos and γ-globin mRNA expression in K562 cells following hemin treatment for 0, 12, 24, or 48 h. (**D**) Western blot analysis of Eos protein levels in untransfected, pcDNA3.1-transfected, and pcDNA3.1-Eos-transfected K562 cells. (**E**) Northern blot analysis of γ-, ε-, and β-globin gene expression in K562 cells before and after 48 h treatment with hemin. K, C, and E represent K562 cells, K562 cells transfected with pcDNA3.1, and K562 cells transfected with pcDNA3.1-Eos, respectively. (**F**) Relative globin mRNA levels in untransfected, pcDNA3.1- and pcDNA3.1-Eos-transfected K562 cells were analyzed by quantitative real-time PCR. The relative level of each globin mRNA is shown as the fold value of the mRNA level in untreated K562 cells. (G) Relative globin mRNA levels before or after hemin-inducted K562 cells were analyzed by quantitative real-time PCR. The relative level of each globin mRNA is shown as fold value of the level of ε-globin mRNA in untreated K562 cells. Each experiment was performed in triplicate, and mRNA levels were normalized to GAPDH or β-actin mRNA expression. Error bars represent one standard deviation. *P<0.05, #*P*<0.01.

Hemin-induced erythroid differentiation of K562 cells was evaluated using the benzidine cytochemical test. Western blot and quantitative real-time PCR indicated that the protein (Figure 1A) and mRNA (Figure 1B) expression of Eos gradually decreased during hemin-induced erythroid differentiation. Conversely, a substantial increase in γ-globin expression was observed during erythroid differentiation of K562 cells (Figure 1A and 1C). The reciprocal association of Eos and γ-globin gene expression following hemin induction in K562 cells supports the hypothesis that the Eos protein might repress γ-globin expression. To study the effect of Eos on γ-globin gene expression, K562 cells were transfected with an Eos expression plasmid (pcDNA3.1-Eos), and overexpression of Eos was confirmed by Western blotting (Figure 1D). Northern blotting revealed that overexpression of Eos significantly downregulated transcription of the γ-globin gene but had little effect on transcription of the α- and ε-globin genes in K562 cells before and after erythroid differentiation (Figure 1E). Quantitative real-time PCR results were consistent with Northern blotting (Figure 1F and 1G). These results support a specific repressive function of Eos on the human γ-globin gene.

### Eos represses γ-globin gene expression in stable μ'LCRAγψβδβ/GM979 transformants

**Figure 2:**
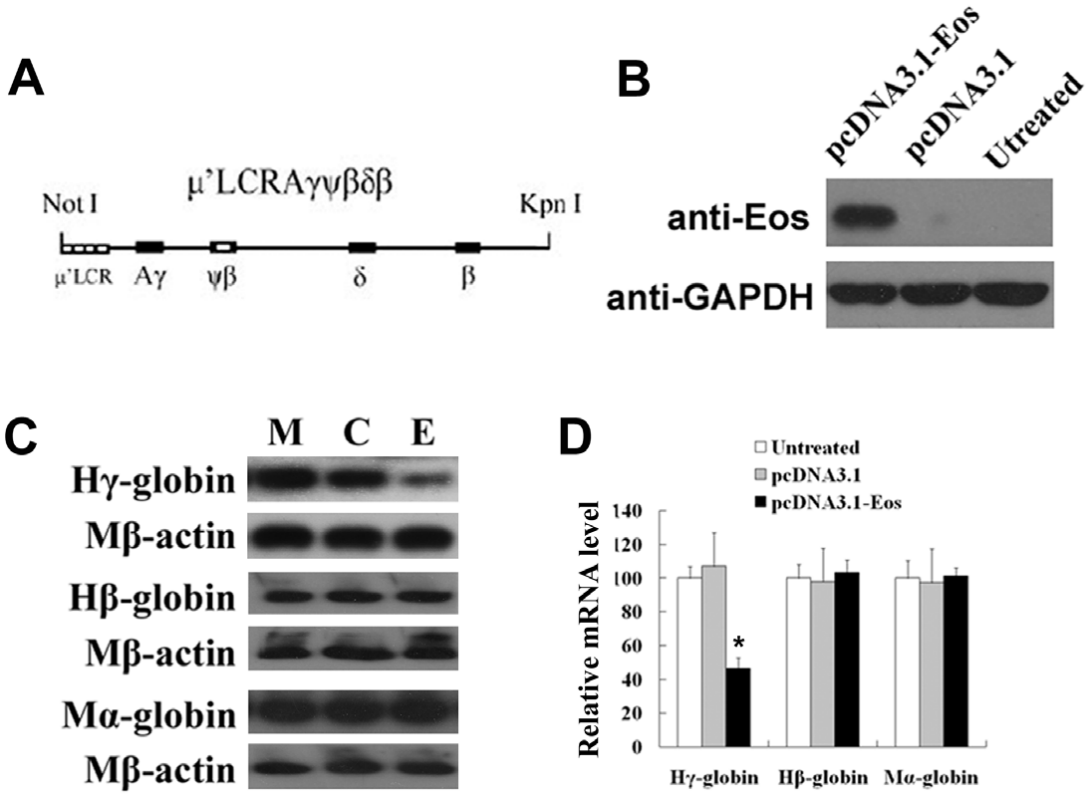
Overexpression of Eos reduces γ-globin gene expression in μ'LCRAγψβδβ/GM979 cells. (**A**) Structural diagram of the integrated construct μ'LCRAγψβδβ, in which a 3.1-kb μ'LCR cassette is linked to a 29-kb fragment from the human Aγ- to β-globin gene. (**B**) Eos protein level was analyzed by Western blot in μ'LCRAγψβδβ/GM979 cells that were untreated and transfected with pcDNA3.1 or pcDNA-Eos. (**C**) The expression levels of human γ-, β-, and murine α-globin genes were analyzed by Northern blot in stable μ'LCRAγψβδβ/GM979 transformants. Each globin mRNA level was normalized to murine β-actin mRNA. M, C, and E represent μ'LCRAγψβδβ/GM979 cells that were untransfected, transfected with pcDNA3.1 (control), and transfected with pcDNA3.1-Eos, respectively. (**D**) Human γ-, β-, and murine α-globin mRNA levels were measured by quantitative real-time PCR. Each PCR analysis was performed in triplicate, and expression levels were normalized to murine β-actin mRNA. Error bars represent standard deviation. *P<0.05.

Because the β-globin gene is not expressed in K562 cells, the effect of Eos on human β-globin cannot be examined in K562 cells. Thus, we used stable MEL GM979 transformants with integration of human β-globin gene cluster. GM979, a MEL cell line which expresses both murine embryonic and adult globins, is an appropriate model system to study human globin gene expression [25]. The human Eos protein is not detected by human Eos antibody in GM979 (Figure S2). A linearized cosmid construct μ'LCRAγψβδβ (Figure 2A), which contained a 3.1 kb μ'LCR cassette, a subset consisting of the impact core sequences of four of the DNaseІ hypersensitive sites, linked to a 29 kb fragment from the human Aγ- to β-globin genes with the natural chromosome arrangement, has been demonstrated a correct developmental expression of human globin genes in transgenic mice [12]. GM979 cells were cotransfected with the linearized cosmid construct μ'LCRAγψβδβ and the pTKneo plasmid by electroporation. Stable transformant cells were elected in medium containing G418. Stable μ'LCRAγψβδβ/GM979 transformants were subsequently transfected with pcDNA3.1-Eos or control vector (pcDNA3.1) respectively. Eos expression then was analyzed by Western blot (Figure 2B). The levels of human Aγ- and β-globin transcripts as well as endogenous murine α-globin transcripts were measured by Northern blot (Figure 2C) and quantitative real-time PCR (Figure 2D). A significant decrease in transcription of human γ-globin was observed in μ'LCRAγψβδβ/GM979 cells overexpressing Eos, whereas human β-globin and murine α-globin transcripts were not significantly affected. These results further supported a specific repressive function of Eos on the expression of human γ-globin gene.

### Identification and validation of functional Eos binding sites within the human β-globin gene cluster

**Figure 3:**
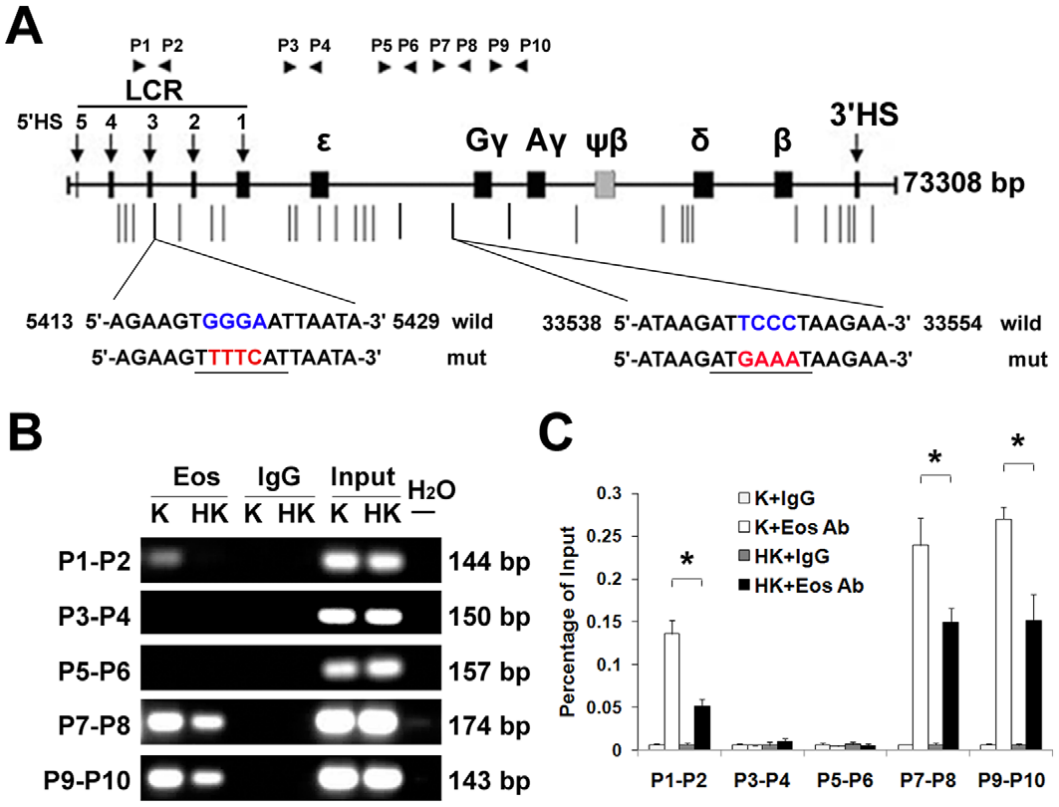
Identification of Eos-binding sites in the β-globin cluster. (**A**) A schematic representation of the human β-globin gene locus. Putative Eos-binding sites (containing WTGGGAA sequence) are shown as vertical lines. Positive ChIP-PCR amplification was obtained using P1–P2, P7–P8, and P9–P10, with P3–P4 and P5–P6 as negative controls. Eos-binding sites and flanking sequences are depicted, and mutated nucleotides are indicated as red in mutation assays. (**B**) ChIP-PCR assays of Eos binding in K562 cells. Amplified fragments using the indicated primers and antibodies demonstrated binding of Eos to the promoter regions of the Gγ- and Aγ-globin genes and to the LCR region in K562 cells. K and HK represent untreated K562 cells and K562 cells treated with hemin for 48 h, respectively. (**C**) Quantitative ChIP analysis of Eos binding to the β-globin gene locus before and after hemin induction in K562 cells. Experimental PCR products were normalized to the PCR products of relevant input DNA. The “K+IgG” and “K+Eos Ab” designations represent the relative occupancies of IgG and Eos antibody, respectively, in the β-globin gene locus before induction. The “HK+IgG” and “HK+Eos Ab” designations indicate the relevant expression after hemin induction. The bar graphs represent averages of three independent ChIP experiments. Error bars depict standard deviation.

To investigate whether Eos represses γ-globin expression by direct association with the human β-globin locus, we searched the human β-globin locus for matches to the Eos binding motif (WGGGAAT). Thirty-two putative Eos binding sites were identified in the β-globin locus (Figure 3A). Chromatin immunoprecipitation (ChIP) was performed using an anti-Eos antibody. Using DNA fragments precipitated with anti-Eos as templates, twenty-eight pairs of primers were designed to amplify the regions containing each of the putative Eos binding sites (Table S1). Of these Thirty-two putative Eos binding sites in the human β-globin cluster, only three discrete regions, which located in the HS3 of LCR, the promoter regions of Gγ- and Aγ-globin genes, were confirmed to be occupied by Eos protein (Figure 3B). A significant reduction of Eos combination at these three binding sites was observed in K562 cells following 48 h of hemin induction, compared with uninduced cells, when the immunoprecipitated DNA was quantified by real-time PCR and compared with the relevant input DNA (Figure 3C). This is consistent with the observed decrease in Eos protein and mRNA expression following hemin induction of K562 cells (Figure 1A and Figure 1B).

**Figure 4:**
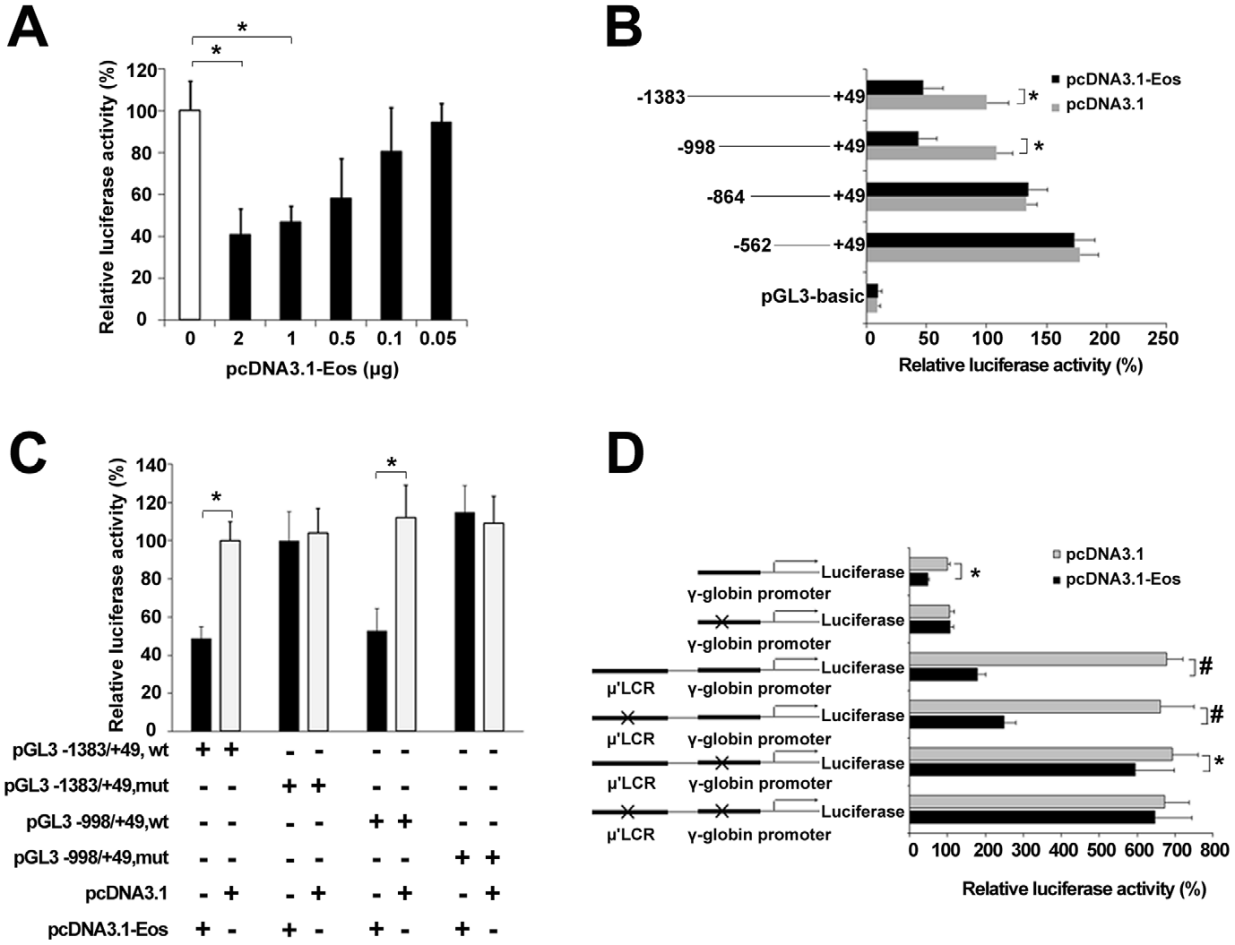
Validation of functional Eos-binding sites in the β-globin cluster using luciferase reporter assays. (**A**) Eos represses γ-globin promoter activity in a dose-dependent manner. Luciferase reporter assays were performed in K562 cells. The recombinant pGL3-basic-1.4 kb γ-globin promoter (−1383/+49) construct was cotransfected into K562 cells with increasing amounts of the pcDNA3.1-Eos expression vector. (**B**) Truncation analysis of γ-globin promoter activity in K562 cells. Cells were cotransfected with pGL3-basic-γ-globin promoter constructs at various lengths and either pcDNA3.1 (i.e., empty vector) or pcDNA3.1-Eos. Promoter/reporter gene constructs with different γ-globin promoter lengths are depicted along the left. (**C**) Mutation analysis of γ-globin promoter activity in K562 cells. pGL3 constructs with different γ-globin promoter regions (−1383/+49 or −998/+49) and with normal or mutated sequences were cotransfected with pcDNA3.1 (i.e., empty vector) or with pcDNA3.1-Eos into K562 cells. (**D**) The luciferase reporter construct consists of the 3.1-kb μ'LCR, the 1.4-kb γ-globin promoter, and the luciferase reporter in the pGL3-basic plasmid (see Materials and Methods). pGL3 constructs with normal or mutated sequences in the LCR or γ-globin promoter were cotransfected with pcDNA3.1 or pcDNA3.1-Eos into K562 cells, and luciferase activities were determined. Luciferase reporter assay data are expressed as percentages of control (i.e., cells transfected with pcDNA3.1 alone) and represent the means ± SE of three separate experiments after correcting for differences in transfection efficiency by pRL-TK activities. Error bars represent one standard deviation. *P<0.05, #*P*<0.01.

To investigate the effect of Eos on the expression of γ-globin gene, a series of dual-luciferase reporter assays were performed in K562 cells. Firstly, a recombinant plasmid including the 1.4-kb γ-globin promoter (pGL3-basic −1383/+49 Gγ/Luc) was constructed and was cotransfected with various concentrations of the Eos expression vector (pcDNA3.1-Eos). Dual-luciferase reporter assays indicated that Eos repressed γ-globin promoter activity in a dose-dependent manner (Figure 4A). When 1 µg of the pcDNA3.1-Eos plasmid was used in luciferase reporter assays, the luciferase activity of pGL3-basic −1383/+49 Gγ/Luc was reduced to about 50% of the activity observed in the absence of the pcDNA3.1-Eos vector. To validate precise sites of silencing elements bound by Eos in the γ-globin promoter, a series of truncated γ-globin promoters (−1383/+49, −998/+49, −864/+49, and −562/+49) linked to a luciferase reporter gene respectively were cotransfected with pcDNA3.1-Eos or pcDNA3.1 empty vector respectively. Deletion analyses revealed that the region between −998 and −864 of the promoter was responsible for the negative effect of Eos overexpression on γ-globin promoter activity (Figure 4B). Mutants containing −1383 to +49 or −998 to +49 of the γ-globin promoter and with a mutation in the Eos binding motif (at approximately position −930) were analyzed by luciferase reporter assay in K562 cells, with cotransfected pDNA3.1-Eos or pDNA3.1. When the Eos binding motif was mutated, overexpression of Eos was not repressive to γ-globin gene expression compared with control (Figure 4C). We generated a pGL3-basic μ'LCR-γ-globin promoter luciferase reporter construct by fusing the 1.4-kb μ'LCR linked to the γ-globin promoter with the luciferase reporter gene in the pGL3-basic vector (Figure 4D). We also generated a series of mutants, including single mutations in the Eos binding site of the LCR or γ-globin promoter and dual-mutations in two of the Eos binding sites. The relative luciferase activity in the K562 cells transfected with the reporter construct containing the μ'LCR was 6.5 times greater than that in the K562 cells transfected with the reporter construct without μ'LCR, and cotransfection with pDNA3.1-Eos reduced luciferase activity significantly. The single mutation of the Eos binding site in LCR partially restored luciferase activity of the LCR-γ-globin promoter construct. The single mutation of the Eos binding site in the γ-globin promoter significantly rescued of luciferase activity. Mutations in both of these sites resulted in a near complete restoration of the luciferase activity. These results suggest that these sites were functional Eos binding sites required for repressive effect of Eos on γ-globin gene.

### The interaction between LCR and the γ-globin promoter is inhibited by Eos

**Figure 5:**
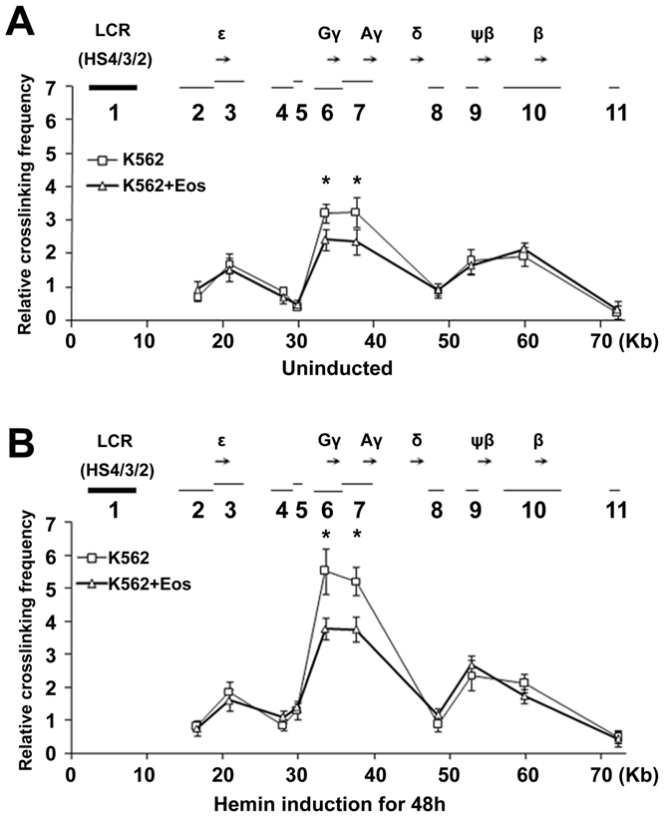
Eos decreases the interaction between the LCR and the γ-globin region promoters. (**A–B**) The human β-globin locus was analyzed by 3C assay in K562 cells that were (**A**) untransfected or transfected with pcDNA3.1-Eos, or (**B**) that were untransfected or transfected with pcDNA3.1-Eos and hemin-induced for 48 h. The relative positions of β-like globin genes are represented as black arrows along the top of each graph. The thick line (1) indicates the position and size of the fixed fragment 1 (LCR HS2/3/4), and the thin lines (2–11) show the position and size of other XbaІ fragments. Data are presented as mean ± SEM (n = 3) cross-linking frequencies between the LCR (HS2/3/4) and differentiation fragments, Error bars represent one standard deviation. *P<0.05.

To ascertain the mechanisms by which Eos inhibits γ-globin gene transcription we performed a chromosome conformation capture (3C) assay in the presence or absence of enforced Eos expression to test if Eos affects the interaction between LCR and the γ-globin promoter in K562 cells. Restriction enzyme XbaІ was used and an XbaІ-digested DNA fragment that includes LCR HS2/3/4 was elected as fixed region, herein referred to as fragment 1. The relative cross-linking efficiency between fragment 1 and other XbaІ fragments then was measured by quantitative real-time PCR. Fragment 1 exhibited significantly higher relative cross-linking efficiencies with fragments 6 that includes Gγ-globin promoter and 7 that includes Aγ-globin promoter than with the other fragments (Figure 5), consistent with the predominant expression of the γ-globin gene compared with the other globin genes in K562 cells. In the presence of enforced Eos, a significant decrease in the interaction between the LCR and the γ-globin promoter was detected both before and after hemin induction (Figure 5). The repressive effect of Eos on γ-globin transcription may be partialy attributed to the inhibition of Eos on the formation of a physical and functional link between the LCR and the γ-globin promoter.

### Eos overexpression reduces γ-globin gene expression in CD34+ HPCs derived from umbilical cord blood

**Figure 6:**
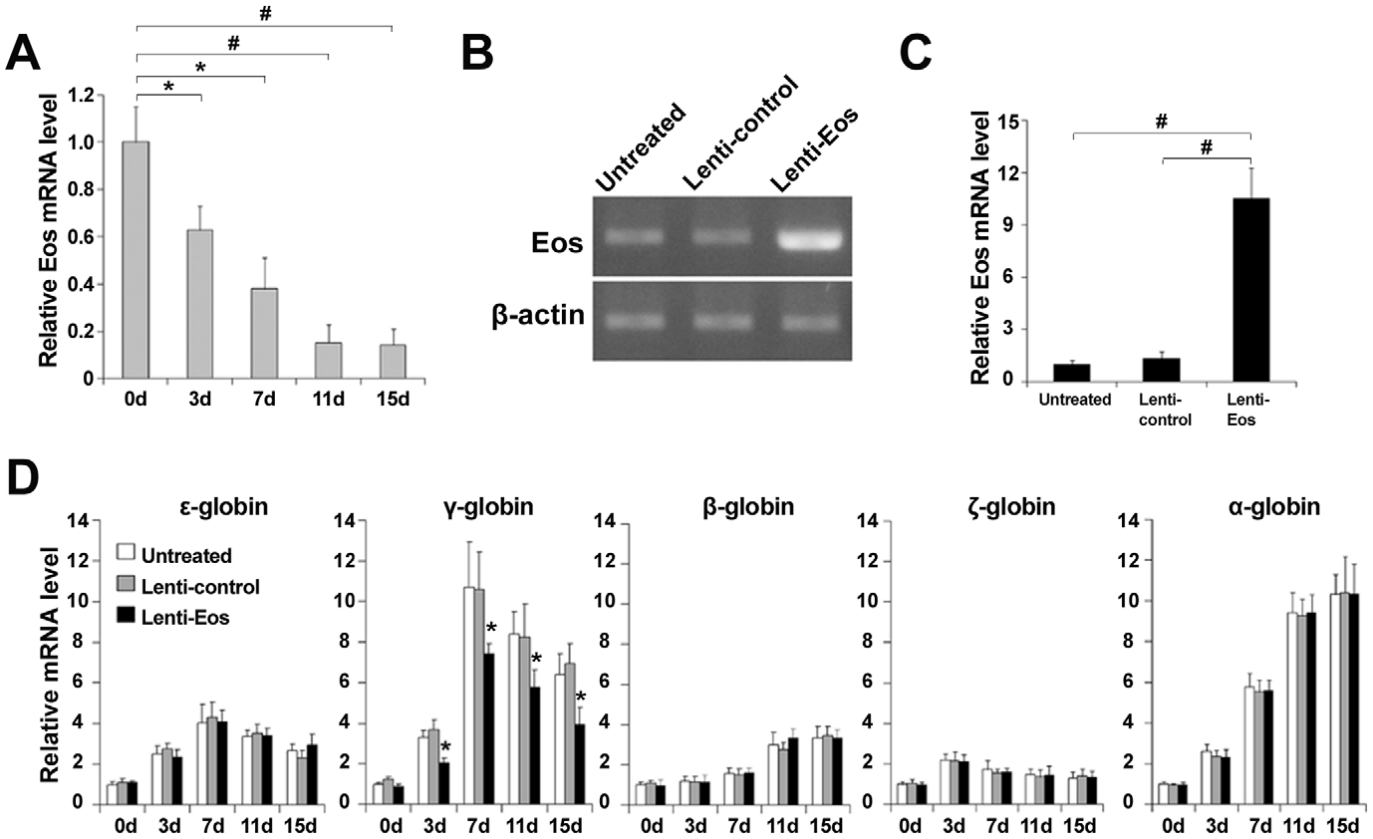
Enforced expression of Eos inhibited the rise in γ-globin gene expression during erythroid differentiation of CD34+ HPCs. (**A**) The Eos mRNA level was analyzed by quantitative real-time PCR during Epo-induced erythroid differentiation of UCB-derived CD34+ HPCs. (**B–C**) The Eos mRNA level was analyzed by (**B**) RT-PCR and (**C**) quantitative real-time PCR in the erythroid induction culture of CD34+ HPCs that were untreated, infected with lentiviruses carrying the pWPXL vector, or infected with recombinant pWPXL-Eos lentiviruses. (**D**) Histograms illustrating globin expression as determined by quantitative real-time PCR in CD34+ HPCs induced into erythroid differentiation for 3, 7, 11, or 15 d. Quantitative real-time PCR experiments were performed in triplicate and were normalized to β-actin mRNA levels. The relative expression of each mRNA is depicted as the fold value in mRNA level compared to untreated CD34+ HPCs. Error bars represent one standard deviation. *P<0.05, #*P*<0.01.

**Figure 7:**
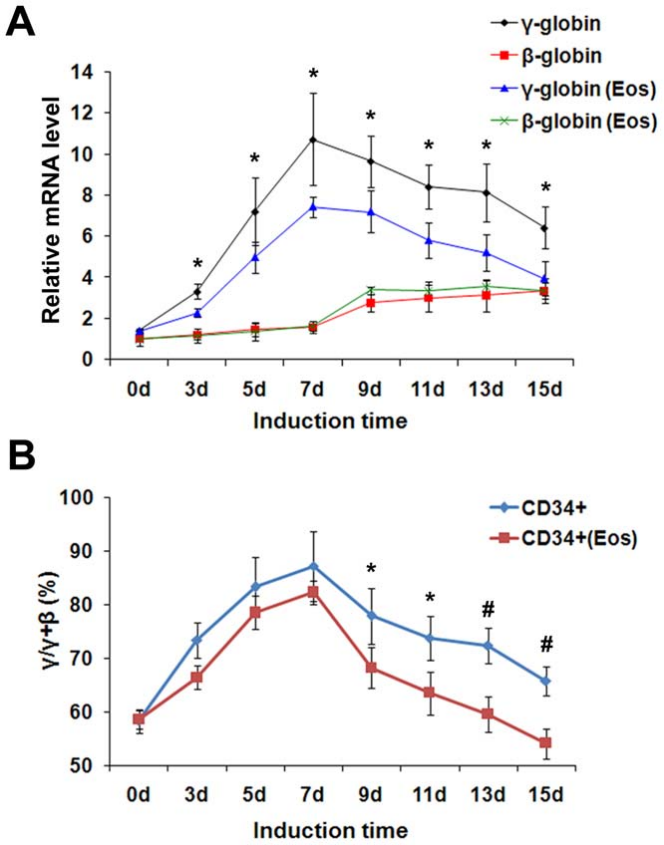
The effect of Eos on γ- to β-globin gene switching during erythroid differentiation. (**A**) Kinetics of γ- and β-globin gene expression during the erythroid maturation of CD34+ HPCs. “Eos” represents CD34+ HPCs infected with lentiviruses carrying Eos. (**B**) Ratio of γ to [γ+β] mRNA during the erythroid maturation of CD34+ HPCs. Quantitative real-time PCR assays were performed in triplicate and were normalized to β-actin mRNA levels. Data were obtained from three independent experiments, and error bars represent standard deviation. *P<0.05, #*P*<0.01.

Human CD34+ HPCs were isolated from human umbilical cord blood (UCB) and were induced to erythroid differentiation using Epo. The Eos mRNA level decreased gradually during Epo-induced erythroid differentiation of CD34+ HPCs, as measured by quantitative real-time PCR (Figure 6A). CD34+ HPCs were infected with the lentivirus control (Lenti-control) and the lentivirus with Eos overexpression (Lenti-Eos) respectively. The high lentivirus transduction efficiency of the CD34+ HPCs was observed through GFP expression (data not shown), and overexpression of Eos mRNA in CD34+ HPCs infected with lenti-Eos was confirmed by conventional RT-PCR (Figure 6B) and quantitative real-time PCR (Figure 6C). Globin mRNA levels in lentivirus-infected CD34+ HPCs at 3, 7, 11, and 15 day of erythroid differentiation also were analyzed by quantitative real-time PCR (Figure 6D). Compared to untransfected and lenti-control-transfected CD34+ HPCs, a significant reduction of γ-globin, but not β-globin, gene expression was observed in CD34+ HPCs transfected with lenti-Eos at each time point of erythroid differentiation (Figure 7A). This suggested enforced Eos expression specifically and continuously inhibited γ-globin gene expression in vivo. Eos appeared to have a minimal effect on γ-globin gene expression prior to Epo induction. This is probably because CD34+ HPCs are a mixture of cells including HSCs and various progenitors, and γ-globin mRNA has minimal expression in CD34+ HPCs.

Additionally, after 7 d of erythroid induction culture when the γ- to β-globin switching appears, a slight increase in β-globin gene expression and an obvious decrease in γ-globin gene expression were detected in lenti-Eos-infected cells compared with controls. Concomitantly, lenti-Eos-infected cells exhibited a slightly more rapid reduction in the ratio of γ to [γ+β]-globin mRNA compared with controls (Figure 7B).

### Enforced expression of Eos inhibits erythroid differentiation

**Figure 8:**
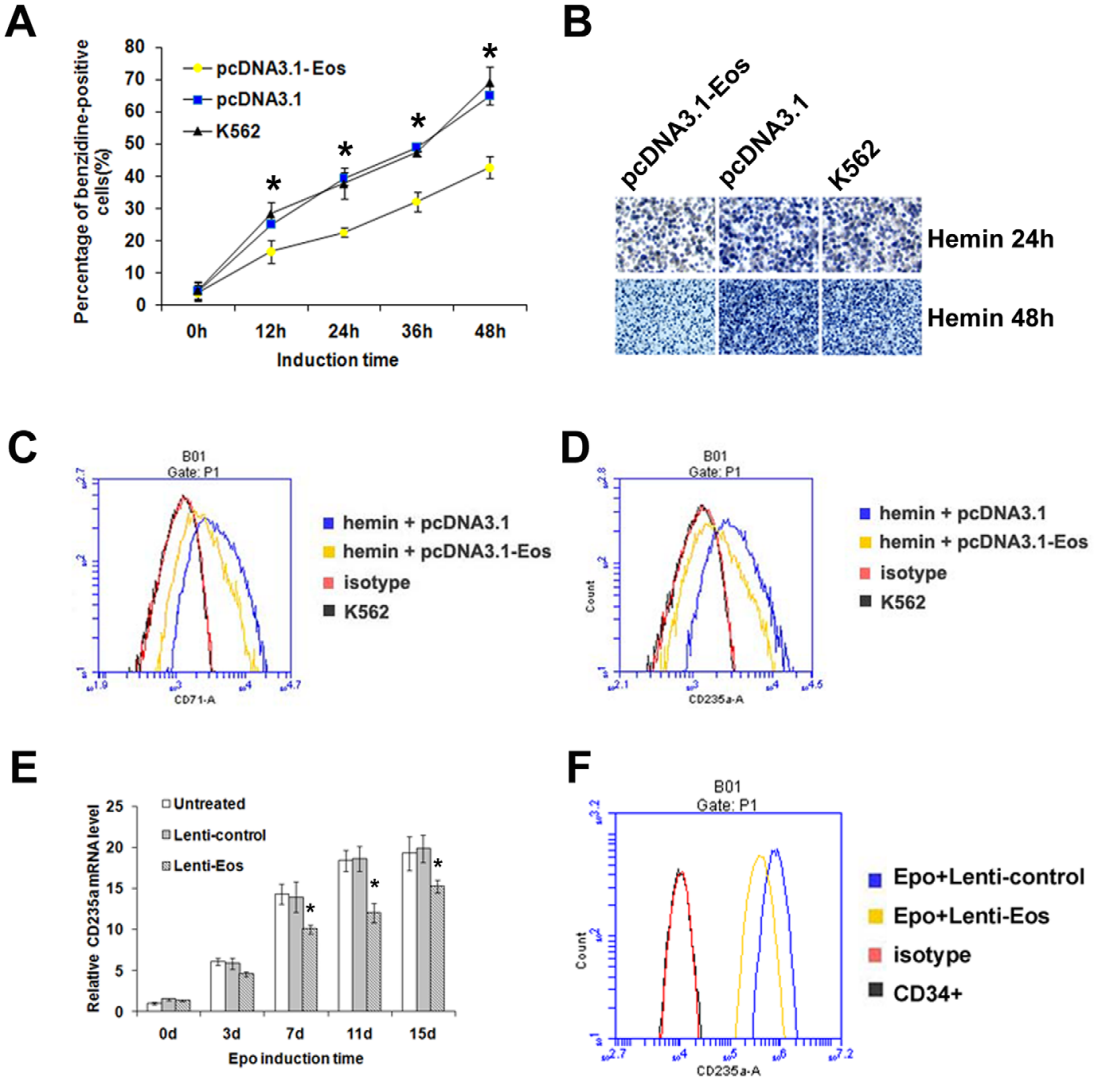
Overexpression of Eos inhibits erythroid differentiation of K562 cells and CD34+ HPCs. (**A**) Benzidine staining of K562 cells that were untransfected, transfected with the control vector (pcDNA3.1), or transfected with the Eos overexpression vector (pcDNA3.1-Eos). The percentage of benzidine-positive cells in each group was counted following hemin induction periods of 0, 12, 24, 36, or 48 h. Data were obtained from three independent experiments, and error bars represent standard deviation. *P<0.05. (**B**) Representative benzidine staining of untransfected and transfected K562 cells that were hemin-induced for 24 or 48 h. (**C–D**) Flow cytometric analysis of K562 cells transfected with control or pcDNA-Eos and treated with (**C**) anti-transferrin receptor (CD71) or (**D**) anti-glycophorin A (CD235a) antibodies after 48 h of hemin induction. (**E**) Quantitative real-time PCR analysis of CD235a mRNA level during Epo-induced erythroid differentiation of CD34+ HPCs. Error bars represent one standard deviation. *P<0.05. (**F**) Flow cytometric analysis of CD235a in K562 cells transfected with lenti-Eos or lenti-control after Epo induction for 15 d.

To examine the role of Eos in erythroid differentiation, untransfected K562 cells and K562 cells transfected with pcDNA3.1 or pcDNA3.1-Eos were induced by hemin. Erythroid differentiation then was evaluated by benzidine staining and flow cytometric analysis of the erythroid markers transferrin receptor (CD71) and anti–glycophorin A (CD235a). A marked decrease in benzidine-positive cells was observed in K562 cells transfected with pcDNA3.1-Eos compared with untransfected cells or cells transfected with control vector (Figure 8A). Representative photos of benzidine-stained K562 cells after 24 or 48 h of hemin induction are showed in Figure 8B. In accordance with benzidine staining results, lower expression levels of CD71 (Figure 8C) and CD235a (Figure 8D) were observed in K562 cells transfected with pcDNA3.1-Eos compared with cells transfected with control vector when the cells were induced by hemin for 48 h.

CD235a also was examined by quantitative real-time PCR and flow cytometric analysis during Epo-induced erythroid differentiation of CD34+ HPCs. Cells infected with lenti-Eos exhibited a reduction in CD235a at each time point during Epo induction compared with controls (Figure 8E). The flow cytometric analysis also demonstrated a decreased expression in CD235a in the erythroid induction culture of CD34+ HPCs infected with lenti-Eos compared with cells infected with lenti-control at day 15 after Epo induction (Figure 8F). These results suggest that overexpression of Eos inhibits erythroid differentiation.

## Discussion

In this study, we identified Eos as a repressor of the γ-globin gene during erythroid differentiation in K562 cells and in CD34+ HPCs. Previous reports have demonstrated that several genes could negatively regulate γ-globin gene expression, whereas stem cell factor (SCF) induces γ-globin gene expression by decreasing COUP-TFII expression [14]. Cohen-Barak et al. reported that Sox 6 binds to the εy-globin gene promoter and represses gene transcription in mice [26]. BCL11A, a multi-zinc finger transcription factor, was originally linked to γ-globin levels in humans by a genome-wide association strategy [27]. The knockdown of *BCL11A* with small interfering RNA resulted in an increase in γ-globin without affecting the expression of other erythroid-specific proteins, such as GATA-1, FOG-1, NF-E2, or EKLF. BCL11A was significantly recruited at HS3 of the LCR and at two sites between the Aγ- and δ-globin genes that were previously implicated in developmental silencing of the γ-globin gene [10]. As a member of the Ikaros family, Ikaros protein is recruited to the human β-globin locus and targets the histone deacetylase, HDAC1, and the chromatin remodeling protein, Mi-2, to the human γ-gene promoters, and thereby contributing to γ-globin gene silencing at the time when the γ- to β-globin gene switching happens [11].

Because a high level of Eos protein expression was detected in K562 cells (Figure S2) and K562 cells have been widely used as a model for the study of globin gene regulation and erythroid differentiation, we examined the effects and mechanisms of Eos on globin gene expression in K562 cells. Since K562 cells do not endogenously express β-globin, we also stably transformed μ'LCRAγψβδβ/GM979 with the human β-globin gene cluster and used erythroid induction cultures of CD34+ HPCs to examine the effects of Eos on globin gene expression. A specific, negative regulatory effect of Eos on the γ-globin gene was demonstrated in all of the three experimental systems.

ChIP-PCR indicated a reduction in Eos proteins bound to the three positive Eos binding sites in the β-globin cluster in the hemin-induced K562 cells compared with uninduced K562 cells (Fig. 3B). This phenomenon could be relevant to the gradual decrease in Eos protein during erythroid differentiation, and it is consistent with the increase in γ-globin gene expression owing to a decrease in Eos repression following hemin induction. Promoter truncation analyses suggested the present of a silencing element between −998 and −864 of the γ-globin promoter that could bind Eos protein. Mutation (TCCC to GAAA) of the Eos binding motif from −929 to −933 in a 1.4-kb of the γ-globin promoter resulted in the nearly complete restoration of luciferase activity in K562 cells expressing a reporter construct and overexpressing Eos. The LCR's influence on γ-globin gene expression was displayed by dual-luciferase reporter assay using a pGL3-basic μ'LCR-γ-globin promoter luciferase reporter construct (Figure 4D). The enforced expression of Eos significantly decreased the luciferase activity. Either a mutation in the Eos binding site in the LCR, or a mutation in the binding site in the γ-globin promoter region resulted in a partially restoration of the luciferase activity. Mutations in both of the two sites resulted in a near complete restoration of the luciferase activity. In the dual-luciferase reporter assays, we speculated that Eos bound to the sites is involved in formation of a repression complex with other protein and the repression complex also reduces the interaction between the LCR and the γ-globin promoter.

We also detected expression of some transcription factors (BCL11A, Ikaros, TR2, TR4, NF-E3, GATA1, EKLF and FKLF), which had been reported to play important roles in γ-globin gene regulation, before and after hemin induction of K562 cells. Real-time PCR assay did not detect significantly change in the expression of these transcription factors when Eos was overexpressed in K562 cells (Figure S3). The results suggested that the γ-globin gene regulation by Eos is not a consequence of the modification in expression of these transcription factors by Eos.

Our 3C assay revealed that HS4, HS3, and HS2 in LCR function as a whole to interact with the γ-globin promoter and regulate gene expression. This is consistent with the previous finding that individual HS core elements interact with the transacting factors bound to the HSs to form a higher-order structure referred to as the LCR “holocomplex” [28]. The repressive effect of Eos on the interaction between the LCR and the γ-globin promoter in K562 cells after hemin induction is more noticeable than before hemin induction (Figure 5). This might be an indirect consequence of Eos influence on K562 cell differentiation.

Our results demonstrated that enforced Eos expression reduced the proportion of benzidine-positive cells compared with control during erythroid differentiation of hemin-induced K562 cells. This was accompanied by decreased expression of CD235a and CD71 (Figure 8), suggesting that Eos may inhibit erythroid differentiation of K562 cells. The increased γ-globin gene expression is an indicator of erythroid differentiation of hemin-induced K562 cells and Epo-induced CD34+ hematopoietic stem/progenitor cells. So the dual effect of Eos on erythroid differentiation and the γ-globin gene regulation are simultaneous and concurrent. However the enforced Eos expression did not significantly reduce transcription of other globin genes during erythroid differentiation in either K562 cells or CD34+ HPCs, which demonstrated a specific negatively regulation effect of Eos on the γ-globin gene. These also suggested that the increase of γ-globin expression during erythroid differentiation is not only a consequence of the effect of Eos on erythroid differentiation. There may be different mechanisms for the repression from Eos on erythroid differentiation and the γ-globin gene expression. In this study, we examined mainly the mechanisms for which Eos regulates the γ-globin gene transcription.

The mammalian β-globin gene locus is a very well-characterized model system for studying long-range chromosomal interactions during erythropoiesis. The LCR is the major structural component of the human β-globin locus, and it is required for high-level globin gene transcription [29]. The human β-globin LCR contains binding sites for several transcription factors, including NFE2, EKLF, GATA-1, and Sp1 [30]. Other reports strongly suggested that contacts between the LCR and various genes of the β-globin locus are developmentally controlled and are required for the LCR to influence the expression rates of individual globin genes [31], [32]. The results of our 3C assay indicated that Eos regulates γ-globin gene expression by inhibiting the interaction between the LCR and the γ-globin promoter (Figure 5). Keys and colleagues examined the role of Ikaros in the assembly of the human β-globin active chromatin hub and subsequent globin gene transcription [20]. Ikaros was involved in human globin gene switching through Ikaros-Eos heterodimers or homodimers [20].

In this study we measured the kinetics of γ- and β-globin genes expression during Epo-induced erythroid maturation of CD34+ HPCs. As shown in Figure 7A, the levels of γ-globin mRNA exhibited marked decreases on the whole and the β-globin mRNA levels exhibited slight increases in the lenti-Eos-infected CD34+ HPCs after day 7 of erythroid induction culture when the conversion of γ- to β-globin gene expression occurs. At the same time, the decline in the ratio of γ to [γ+β]-globin mRNAs also appeared to be a little more rapid in the Eos-virus-infected cells compared with controls (Figure 7B). These results suggest that enforced Eos expression had a minor effect on γ- to β-globin switching during erythroid differentiation of HPCs.

In conclusion, the present study suggests that Eos contributes significantly to the transcriptional regulation of γ-globin during erythroid differentiation of K562 cells and UCB-derived CD34+ HPCs.

## Materials and Methods

### Cell lines, cell culture, and erythroid induction of K562 cells

Chronic myelogenous leukemia cell line K562 was purchased from the Cell Center of The Institute of Basic Medical Science, Chinese Academy of Medical Science. Human embryonic kidney cell line 293TN was purchased from System Biosciences (SBI, CA, USA). The mouse erythroleukemia cell line MEL GM979 was a gift from Dr. Stamatoyannopoulos at University of Washington. All of the cells were cultured at 37°C and 5% CO_2_ in RPMI 1640 medium or Dulbecco's Modified Eagle Medium (GIBCO, NY, USA), supplemented with 10% fetal bovine serum (GIBCO), 2 mM L-glutamin, 100 U/mL penicillin, and 100 µg/mL streptomycin (Invitrogen, CA, USA). K562 cells were induced into erythroid differentiation by incubation in medium containing 50 µM hemin (Sigma-Aldrich, Germany). Cells were harvested at each desired time point (0, 24, 48, and 72 h).

### RNA isolation and quantitative real-time PCR analysis

Total RNA was isolated from cells harvested using TRIzol reagent (Invitrogen) according to the manufacturer's instructions. RNA was reverse-transcribed to cDNA using the M-MLV reverse transcriptional system (Invitrogen). Quantitative real-time PCR were performed using an iQ5 Real-Time PCR Detection System (Bio-Rad, California, USA) and SYBR Premix Ex Taq kit (Takara, Dalian, P. R. China). Primers used in quantitative real-time PCR are listed in Table S2.

### Plasmid constructs

The sequences encoding Eos were amplified by PCR from cDNA derived from K562 cells and cloned into the pcDNA3.1(+) expression vector (Invitrogen) or the pWPXL retroviral expression vector (Addgene). The primers Eos-F (5′- TGCCTGCGAAATGACGG -3′) and Eos-R (5′- AGGGCACAAGAGGTATGGAGTA -3′) were used for PCR amplification.

Promoter region of the γ-globin gene (−1383 to +49 relative to the transcription start site) was amplified from human genomic DNA and cloned into the luciferase reporter vector pGL3-basic (Promega, Madison, WI, USA). A series of truncated γ-globin promoter regions including −562 to +49, −864 to +49, and −998 to +49, were cloned into the pGL3-basic vector as described previously [33]. The 3.1-kb micro-LCR (μ'LCR) sequence was amplified from the cosmid construct μ'LCRAγψβδβ [34] by PCR and was inserted upstream of the γ-globin promoter in the pGL3-basic plasmid. Mutations in the putative Eos binding sequence of the construct plasmid were introduced using PCR-based site-directed mutagenesis. The bases TTTC replaced GGGA at LCR region and GAAA replaced TCCC in the approximately position −930 of the γ-globin gene promoter (Fig. 3A). All plasmids were prepared using the Plasmid Maxi Kit (Qiagen, CA, USA). All constructs were sequence-verified.

### Northern blot and Western blot

Northern blot analysis of globin mRNAs was performed as described previously [35]. Briefly, T4 polynucleotide kinase and γ-^32^P ATP were used to 5′ end-label ssDNA probes (NEB). Probe sequences are listed in Table S3. Western blot analysis was performed as described previously [24]. The following primary antibodies were used: anti-Eos (Santa Cruz Biotechnology, Inc., CA, USA), anti-γ-globin (Santa Cruz), anti-β-actin (Proteintech, Group Inc., Chicago, IL), and anti-GAPDH (Proteintech). HRP-conjugated secondary antibodies were used. Immunoblots were quantified using AlphaEaseFC software.

### Cell transfection and luciferase reporter assay

GM979 cells were stably transformed with μ'LCRAγψβδβ as described previously [36]. Briefly, 2×10^7^ cells were cotransfected with linearized cosmid μ'LCRAγψβδβ and linearized plasmid pTKneo in HEPES buffered saline by electroporation at 250 V and 960 µF. Stable GM979 transfectants were selected in medium containing 130 µg/mL G418 for 2 weeks.

For the dual-luciferase reporter assay, K562 cells were seeded in 24-well plates and cotransfected with plasmid pcDNA3.1-Eos or with pcDNA3.1 and the luciferase reporter plasmid (pGL3-basic-based construct and pRL-TK plasmid), respectively, using Lipofectamine LTX reagent (Invitrogen) according to the manufacturer's instructions. The transfection medium was replaced with complete medium after 6 h, and cells were cultured for 48 h. Cells then were lysed using Passive Lysis Buffer (Promega), and luciferase activities were measured with a Modulus Microplate Luminometer (Turner Biosystems, CA, USA) using the Dual-Luciferase Reporter Assay System (Promega) according to the manufacturer's instructions.

### Chromatin immunoprecipitation-PCR (ChIP-PCR)

Chromatin immunoprecipitation (ChIP) assays were performed essentially as previously reported [37]. Briefly, uninduced and hemin-induced K562 cells were harvested and fixed in 1% formaldehyde (Sigma-Aldrich, Deisenhofen, Germany) at room temperature for 10 min and quenched for 5 min with glycine. Cells were lysed and sonicated to obtain chromatin fragments that were approximately 500–1000 bp in length. ChIP was performed using the EZ-ChIP Chromatin Immunoprecipitation Kit (Millipore, MA, USA) with minor modifications to the manufacturer's instructions. A rabbit polyclonal anti-Eos antibody (Santa Cruz) was used as the immunoprecipitating antibody, and rabbit IgG (Santa Cruz) was used as the control. Input and immunoprecipitated DNA were amplified by PCR using primers listed in Table S1. Quantitative real-time PCR was performed as described previously [38], [39]. Immunoprecipitated DNA was amplified using SYBR green dye on a Bio-Rad iQ5 Real-Time PCR Detection System, and experimental PCR products quantified by comparison with the PCR products of a dilution series of relevant input DNA.

### Chromosome conformation capture (3C) assay

The 3C assay was performed as described previously [40], [41] with minor modifications. K562 cells (1×10^8^) were harvested and crosslinked with 1% formaldehyde at room temperature for 10 min and quenched with glycine to a final concentration of 0.125 M. After cells were lysed, SDS was added to a final concentration of 0.1%, and the reaction was incubated at 37°C for 10 min. Triton X-100 then was added to 1%. DNA was digested with XbaІ (NEB) overnight at 37°C. The restriction enzyme was inactivated with the addition of 1.6% SDS, and the digested DNA was incubated at 65°C for 20 min. The reaction was diluted to 2.5 ng/µL DNA, and Triton X-100 was added to 1%. DNA was ligated for 4–5 h at 16°C using T4 ligase (NEB). Cross-links were reversed by overnight incubation in 5 µg/mL proteinase K at 65°C. DNA was purified by phenol-chloroform extraction and ethanol precipitation. DNA concentrations were measured using the NanoDrop 2000 Spectrophotometer (Thermo Fisher Scientific Inc., Bremen, Germany) and were diluted for quantitative real-time PCR.

To generate the control template with detectable amounts of randomly ligated DNA fragments, PCR fragments from ligation products, which spanned each restriction sites, were purified from the agarose gel to enrich for the ligation products of interest. Equimolar concentrations of the PCR fragments were mixed, digested with XbaІ, and ligated. The ligated fragments were purified by phenol extraction and ethanol precipitation and diluted to an appropriate concentration. Purified fragments (300 ng) were mixed with the same amount of digested and randomly ligated genomic DNA. The mixture was used as control sample for quantitative real-time PCR. The experimental PCR products were quantified by comparison with the PCR products of relevant control. The 3C primers used in this study are listed in Table S4.

### Assay of erythroid differentiation: benzidine staining and flow cytometry

Erythroid differentiation of K562 cells was scored by benzidine staining as reported previously [42]. Flow cytometry was carried out according to standard protocols, and samples were analyzed using a C6 flow cytometer (Accuri Cytometers, MI, USA).

### Isolation and erythroid induction of CD34+ HPCs and recombinant lentivirus generation/infection

Human UCB samples were obtained with informed consent from normal, full-term deliveries. CD34+ HPCs were purified from normal UCB using magnetic-activated cell sorting (MACS) technology according to the manufacturer's recommendations (Miltenyi Biotec, Bergisch-Gladbach, Germany). CD34+ HPCs were cultured in IMDM supplemented with 30% fetal bovine serum (GIBCO), 1% BSA, 100 µM 2-mercaptoethanol (2-ME) (Sigma), 2 ng/mL recombinant human interleukin-3 (IL-3), 100 ng/mL recombinant human stem cell factor (SCF) (PeproTech, London, UK), 2 U/mL recombinant human Epo (R&D Systems, MN, USA), 60 mg/mL penicillin, and 100 mg/mL streptomycin. Cells were harvested every 2 d during erythroid induction.

The expression plasmid pWPXL-Eos was cotransfected with packaging plasmids into 293TN cells using Lipofectamine with Plus Reagent (Invitrogen). The packaging kit was purchased from System Biosciences and operated according to the manufacturer's instructions. Recombinant lentivirus particles (i.e., lenti-Eos and lenti-control) were harvested and added to the medium of CD34+ HPCs in culture. Lentivirus-infected CD34+ HPCs were washed with PBS and induced to erythroid differentiation with Epo for 2 weeks.

### Statistics

Each set of experiments was repeated at least in triplicate, and standard error values were calculated. Data were analyzed using Student's two-tailed *t*-test, and *P*-values less than 0.05 were considered significant.

## Supporting Information

Figure S1 **Schematic representation of Eos protein structure.** The black ovals represent the zinc finger regions, and N-terminal DNA-binding zinc fingers can bind to DNA binding motif sequences (WGGGAAT).
(TIF)

Figure S2 **Western blot analysis of Eos expression in some human cell lines and MELGM979.**
(TIF)

Figure S3 **Quantitative real-time PCR analysis of mRNA levels of eight transcription factors in untransfected, pcDNA3.1-transfected and pc3.1-Eos-transfected K562 cells.** (**A**) Before hemin treatment. (**B**) After 48 h with hemin treatment. Each real-time PCR experiment was performed in triplicate and mRNA level was normalized to β-actin mRNA expression. The relative expression of each mRNA was shown as the fold values of mRNA levels in untreated K562 cells.
(TIF)

Table S1 **Primers used for ChIP-PCR.**
(DOC)

Table S2 **Primers used for real-time PCR.**
(DOC)

Table S3 **Probes used for Northern blot.**
(DOC)

Table S4 **Primers used in the 3C assay.**
(DOC)
