# Supplementary figures and images for: Eos Negatively Regulates Human γ-globin Gene Transcription during Erythroid Differentiation
Source: PLoS One. 2011 Jul 28;6(7):e22907. doi: 10.1371/journal.pone.0022907 (PMC3145782; doi:10.1371/journal.pone.0022907)

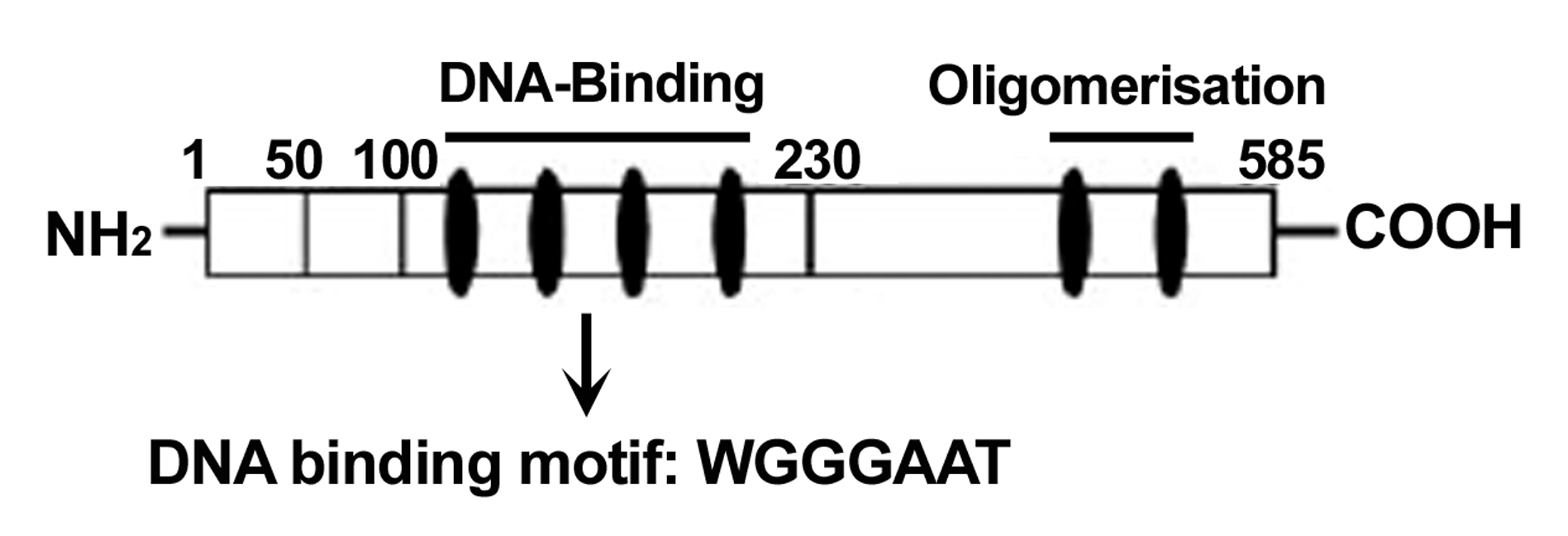

Supplement: Figure S1 — Schematic representation of Eos protein structure. The black ovals represent the zinc finger regions, and N-terminal DNA-binding zinc fingers can bind to DNA binding motif sequences (WGGGAAT). (TIF) [file pone.0022907.s001.tif]

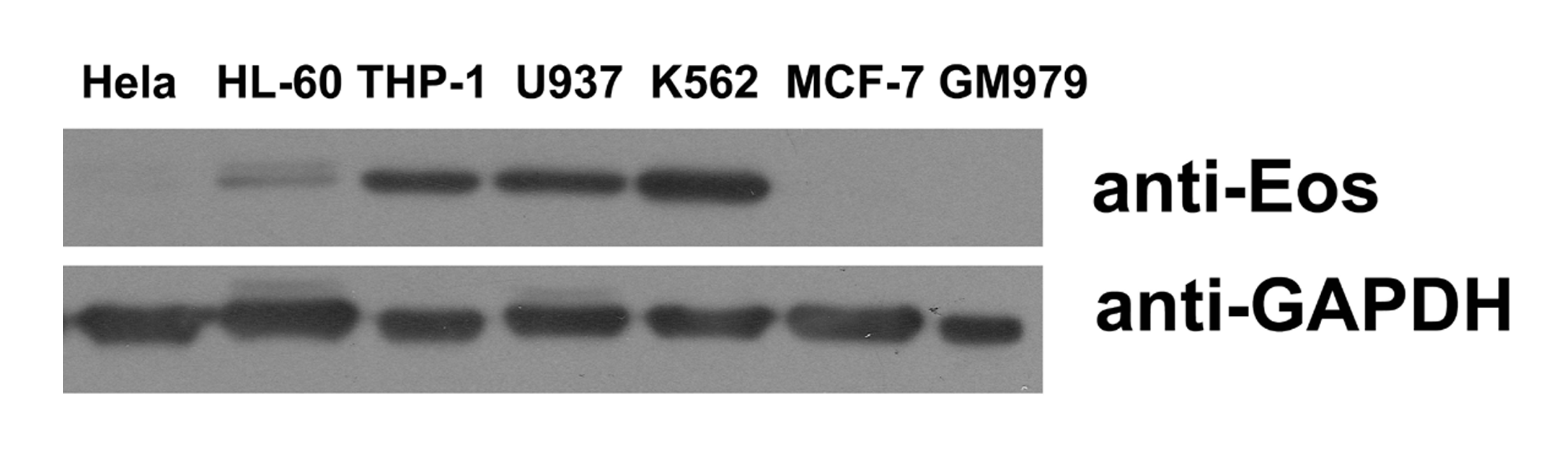

Supplement: Figure S2 — Western blot analysis of Eos expression in some human cell lines and MELGM979. (TIF) [file pone.0022907.s002.tif]

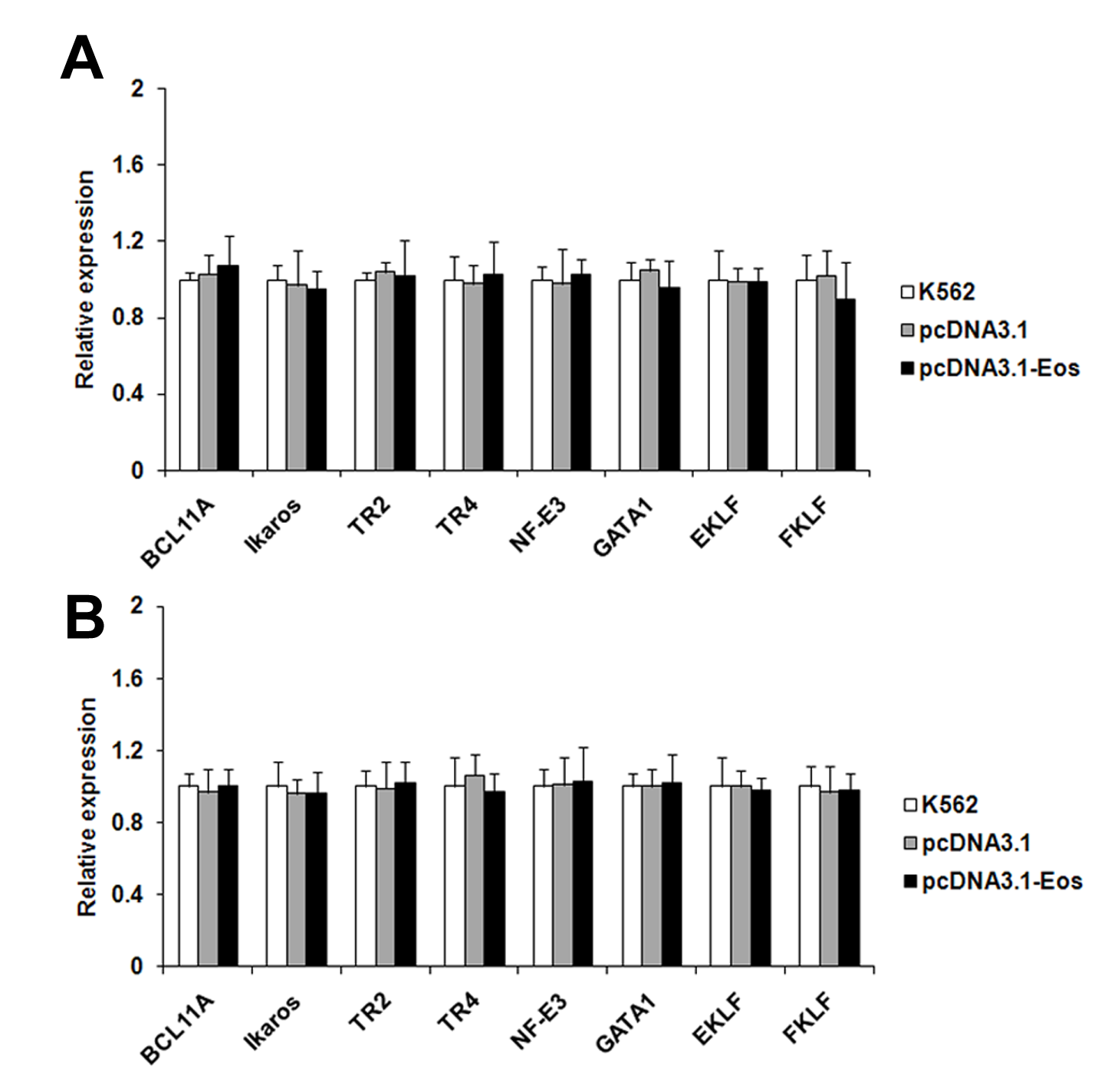

Supplement: Figure S3 — Quantitative real-time PCR analysis of mRNA levels of eight transcription factors in untransfected, pcDNA3.1-transfected and pc3.1-Eos-transfected K562 cells. (A) Before hemin treatment. (B) After 48 h with hemin treatment. Each real-time PCR experiment was performed in triplicate and mRNA level was normalized to β-actin mRNA expression. The relative expression of each mRNA was shown as the fold values of mRNA levels in untreated K562 cells. (TIF) [file pone.0022907.s003.tif]
