# Supplementary material for: Eos Negatively Regulates Human γ-globin Gene Transcription during Erythroid Differentiation
Source: PLoS One. 2011 Jul 28;6(7):e22907. doi: 10.1371/journal.pone.0022907 (PMC3145782; doi:10.1371/journal.pone.0022907)
Supplement: Table S1 — Primers used for ChIP-PCR. (DOC) [file pone.0022907.s004.doc]

**Table S1.** Primers used for ChIP-PCR

| **Primers for ChIP-PCR （5’-3’）** | | |
| --- | --- | --- |
| Name | Sequences | HUMHBB |
| EOSP1F | GAGTTCTGGCACTTGTCACTATGC | 3118, 3140 |
| EOSP1R | CCAAGCCCTTGCTTTTGCT |
| EOSP2F | CCATCATTGAATAAATCCCTATAGG | 3338 |
| EOSP2R | GCATATGATGGGGTAGCAGAC |
| EOSP3F | CTGAACCCTGCTGAGATGATC | 4018 |
| EOSP3R | TTACTCATCCCATCACGTATGC |
| EOSP4F | GGAAACAAGATGGTGAAGGG | **5418**  **(p1/p2)** |
| EOSP4R | AATATCATAAAATGGCACCTGA |
| EOSP5F | TAGAAGGGTACTTTCACAGGAACA | 7206 |
| EOSP5R | GGCAGCAGATTCAGCTTATTTT |
| EOSP6F | AGCCTCCAGAGCGAGACTCT | 10931 |
| EOSP6R | GGATGCTGACCAGAGGCTT |
| EOSP7F | CCTTCACCGAATCTCATCCTAC | 11900 |
| EOSP7R | AGTACCCATAGATGATGAGGAACC |
| EOSP8F | GCAATACTCACAAAGTAGCCAGTG | 18827, 18872 |
| EOSP8R | GGACTAAAGCCTTAATCCCAAAGT |
| EOSP9F | GCCTGTGGAGCAAGATGAATG | **19655**  **P3/P4** |
| EOSP9R | GGGTAAACAACGAGGAGTCTATGA |
| EOSP10F | CATAGGAAGAACCAAGAGCTTCC | 21235, 21244 |
| EOSP10R | CGGTGCAGTAAAAACTGCCT |
| EOSP11F | CTAGTGAGATTGCTGGATTGTATGAT | 23416 |
| EOSP11R | CAGATCCTTGTGAGGATATGGAG |
| EOSP12F | ACTTGCTTGTTTATTGGGCTCT | 24355 |
| EOSP12R | TGCCATTCTCATAGATGGGTAGA |
| EOSP13F | GGTTAATCTTGCTAATGGTCTATCAGT | 25687 |
| EOSP13R | AGGGAATAACTAAGATAAGAGGAGAACT |
| EOSP14F | GTATAAGGTGTAAGGAAGGGGTAC | **27131**  **(p5/p6)** |
| EOSP14R | GCCTCAGAAATAATGCCACA |
| EOSP15F | AAAACAGTCCCTGCCTCTTAA | **33543**  **(p7/p8)** |
| EOSP15R | GTGTAGCTCTTCTATGCTCGGT |
| EOSP16F | ACCCTTGCCTCTTTAGAGTTG | **38481**  **(p9/p10)** |
| EOSP16R | GATGATGGTAGAATGTTCTTTGG |
| EOSP17F | GGGGCAACTGTTCACTGGTA | 43831 |
| EOSP17R | CCTCAGAGGAAACTTCAGGTCAT |
| EOSP18F | GTATTCATCACACTCTTGGATTCAC | 51621 |
| EOSP18R | CTGAGTCTGAGGTGCCTATAGGA |
| EOSP19F | GATCTTCATTCTGGGTCTAATTCC | 53972 |
| EOSP19R | CAAAGTAGATTTACCATATTCAGCCA |
| EOSP20F | CAAAAGAAGGAGGAAGCAAGC | 54315 |
| EOSP20R | TTAGGCTAATATAGTGGAATGTATCTTAGAGT |
| EOSP21F | GATGCGGTGGGGAGATATG | 54606 |
| EOSP21R | GCCTTTTATGCTGGTCCTGTC |
| EOSP22F | GCTAGTTAAGTACATAGAGGATGTGTGTG | 55999 |
| EOSP22R | TTCTGAGAAACTGAGCCAACAC |
| EOSP23F | TGAGCATCTGGATTCTGCC | 63782 |
| EOSP23R | AGTGTATTTTCCCAAGGTTTGAAC |
| EOSP24F | TGCATGAGCCGAAGCAG | 67287, 67292 |
| EOSP24R | CAGTATTAGGGCGGGAGTGAC |
| EOSP25F | TTCTACCAGAGGTACAAGGAGGAG | 70674 |
| EOSP25R | GGCTTTGGTATCAGGATGATG |
| EOSP26F | GACAAACCCACAGCCAATATC | 71118 |
| EOSP26R | CTGCCTGATTGCCCTGG |
| EOSP27F | GCCGCATATCTACAACTATCCG | 71954 |
| EOSP27R | TGTATAAGGTGTAAGGAAGGATCCA |
| EOSP28F | TGATGAGTTCATGTCCTTTGTAGG | 72986 |
| EOSP28R | CCAGAGTGTGATGTTCCTCTTC |
