# Supplementary material for: Eos Negatively Regulates Human γ-globin Gene Transcription during Erythroid Differentiation
Source: PLoS One. 2011 Jul 28;6(7):e22907. doi: 10.1371/journal.pone.0022907 (PMC3145782; doi:10.1371/journal.pone.0022907)
Supplement: Table S2 — Primers used for real-time PCR. (DOC) [file pone.0022907.s005.doc]

**Table S2.** Primers used for real-time PCR

| **Primers for Real-time PCR （****5’-3’）** | |
| --- | --- |
| γ-globin F | GCAGCTTGTCACAGTGCAGTTC |
| γ-globin R | TGGCAAGAAGGTGCTGACTTC |
| β-globin F | GTCTACCCTTGGACCCAGAGGTTC |
| β-globin R | TGAGCCAGGCCATCACTAAAG |
| ε-globin F | CAGCTGCAATCACTAGCAAGC |
| ε-globin R | AGACGACAGGTTTCCAAAGC |
| α-globin F | GGTCAACTTCAAGCTCCTAAGC |
| α-globin R | GCTCACAGAAGCCAGGAACTTG |
| ξ-globin F | TGAGCGAGCTGCACGCCT AC |
| ξ-globin R | GTACTTCTCGGTCAGGACAGA |
| GAPDH F | TCAACGACCACTTTGTCAAGCTCA |
| GAPDH R | GCTGGTGGTCCAGGGGTCTTACT |
| β-actin F | CTGGCACCACACCTTCTACA |
| β-actin R | AGCACAGCCTGGATAGCAAC |
| Eos F | GCAAGGGAAGGATAATCTGGAG |
| Eos R | TGAGTCCCCGCTACTTTCAC |
| Mα-globin F | GAAGAAACCATGGTGCTCTCTG |
| Mα-globin R | CTTGACCTGGGCAGAGCC |
| M GAPDH F | CATGGCCTTCCGTGTTCCTA |
| M GAPDH R | GCGGCACGTCAGATCCA |
| CD235a F | GGCTGGTGTTATTGGAACGATC |
| CD235a R | GAGGTTTTACATCAGATGGGCTTT |
| BCL11A F | GCTCAAAAGAGGGCAGACG |
| BCL11A R | TGGGCACAGGCATAGTTG |
| Ikaros F | CGGCTTTGTCGGGAGTTG |
| Ikaros R | GCCCTTCTGGGTGAATGAG |
| TR2 F | TGGAGACAAAGCATCAGGACG |
| TR2 R | GCAGTATTGACAGCGGTTTCG |
| TR4 F | CCTTGCCAACCTAAGTGAATC |
| TR4 R | ACTGGTGTCTATCCCATCTGC |
| NF-E3 F | AGCACCATCGCAACCAGTG |
| NF-E3 R | ATCCGGACAGGTACGAGTGG |
| GATA1 F | GGACCTGCACTGCCTTCATC |
| GATA1 R | ACACACTCCCTGGCCTCACA |
| EKLF F | CCAAGAGCTCCCACCTGAAG |
| EKLF R | CCCGTGTGTTTCCGGTAGTG |
| FKLF F | GGAAGCTGTCGAGGCTCTTG |
| FKLF R | GATGGCTCCACGAGATCAGG |
