# Supplementary material for: Eos Negatively Regulates Human γ-globin Gene Transcription during Erythroid Differentiation
Source: PLoS One. 2011 Jul 28;6(7):e22907. doi: 10.1371/journal.pone.0022907 (PMC3145782; doi:10.1371/journal.pone.0022907)
Supplement: Table S3 — Probes used for Northern blot. (DOC) [file pone.0022907.s006.doc]

**Table S3.** Probes used for Northern blot

| **Probe Sequences for Northern blot （5’-3’）** | |
| --- | --- |
| Hγ-globin | GAACCTCTGGGTCCATGGGTAGACAAC |
| Hβ-globin | CAAAGAACCTCTGGGTCCAAGGGTAG |
| Hε-globin | CACATGCAGCTTGTCACAGTGCAG |
| Hα-globin | GAGCCGTGGCTCAGGTCGAAGTG |
| Hβ-actin | GTACATGGCTGGGGTGTTGAAGG |
| Mα-globin | GTAGGTCTTGGTGGTGGGGAAGCTAG |
| Mβ-actin | GAGCATCGTCGCCCGCGAAG |
