# Supplementary material for: Eos Negatively Regulates Human γ-globin Gene Transcription during Erythroid Differentiation
Source: PLoS One. 2011 Jul 28;6(7):e22907. doi: 10.1371/journal.pone.0022907 (PMC3145782; doi:10.1371/journal.pone.0022907)
Supplement: Table S4 — Primers used in the 3C assay. (DOC) [file pone.0022907.s007.doc]

**Table S4**  Primers used in the 3C assay

| **Primers for 3C （5’-3’）** | |
| --- | --- |
| Xba-1 | CCTGCAGCAAGAATGATTGC |
| Xba-2 | CAATACTCTGAAGTAGTTCAGAATGTGC |
| Xba-3 | TCTAGCTCTCTTAAGGAGTGCATTG |
| Xba-4 | TTTTACACTGTTGATGGGTGTGTA |
| Xba-5 | GAAACAATGGATGAATGGATGC |
| Xba-6 | GGCTGAGACCCAGTGGC |
| Xba-7 | AAGAAACTGCAGAGGACTAACTGG |
| Xba-8 | CTTACACCTCCAAAGAGTACCTCC |
| Xba-9 | TTTAAACTGCAGCAATAGTTGTCC |
| Xba-10 | GGTGGGAGAATCAGGAAACTATTAC |
| Xba-11 | GGGACTGTAAACTAGTTCAACCATT |
